# Prevalence of Herpes Simplex Virus 2 (HSV-2) infection and associated risk factors in a cohort of HIV negative women in Durban, South Africa

**DOI:** 10.1186/s13104-016-2319-5

**Published:** 2016-12-12

**Authors:** Brodie Daniels, Handan Wand, Gita Ramjee, the MDP Team

**Affiliations:** 1 HIV Prevention Research Unit, Medical Research Council, Durban, South Africa; 2 The Kirby Institute, Sydney, Australia

**Keywords:** HSV-2, Women, Risk factors, South Africa

## Abstract

**Background:**

Herpes Simplex Virus 2 (HSV-2) is one of the most common sexually transmitted infections (STIs) worldwide and is a risk factor for the acquisition and transmission of other STIs, including HIV. We determined the prevalence and predictors of HSV-2 infection among women screened for a HIV prevention trial in Durban, South Africa. Univariate and multivariate logistic and Cox regression models were used to determine the correlates and predictors of HSV-2 infection at enrolment and seroconversion during the study respectively.

**Results:**

Prevalence of HSV-2 at screening was 65% and crude incidence was 22.3 per 100 person-years (PY) (95% CI 20.4–24.3). The HIV seroconversion was significantly higher among those testing positive for HSV-2 at baseline compared to women who were negative [8.7 per 100 person years (PY) versus 5.2 per 100 PY; (p < 0.001)]. In univariate analysis, age was determined to be the most significant predictor for HSV-2 diagnosis, while co-infection with syphilis was also a significant predictor, while age and co-infection with syphilis remained the two most significant predictors of having HSV-2 in multivariate analysis at baseline. Consistent with these results, along with HIV seroconversion, age was also identified as a significant predictor for incidence of HSV-2.

**Conclusion:**

Given the unacceptably high prevalence and incidence rates of HSV-2 infection reported here, HSV-2 and general STI education needs to be reinforced in these communities, with a focus on condom education for prevention. HSV-2 has emerged as the most prevalent STI which is most often asymptomatic and unrecognized, and which increases women’s risk of acquiring other STIs, including HIV.

## Background

Herpes Simplex Virus 2 (HSV-2) is a chronic, sexually transmitted infection (STI) which is infectious during both its symptomatic and asymptomatic periods [1]. This asymptomatic nature of HSV-2 infection assists in the spread of the infection in the general population [2]. It has been estimated that around 500 million people are currently infected with HSV-2 worldwide and that approximately 20 million new cases occur each year. In European countries, the prevalence of HSV-2 fluctuates significantly between countries, varying from approximately 5% for England and Spain, approximately 20–30% for countries such as Germany and Sweden up to 40% for Turkey [3]. Considerably higher rates of HSV-2 have been observed in sub-Saharan Africa (SSA), with age‐adjusted prevalence in adults ranging from 10 to 50% in men and 30 to 80% in women [4]. Cross sectional studies in SSA have shown a prevalence of 5–53% in 13–24 year old men and women, respectively [5]. In Uganda, men aged 15–19 years old had 10% prevalence while 20–24 year old men had a 27% prevalence. Ugandan women, showed a 35% prevalence among 15–19 year olds versus a 74% prevalence in women aged 20–24 years old [5]. In South Africa, studies have shown a 31% prevalence of HSV-2 in women aged 15–26 [6], while an 84% prevalence was shown among female commercial sex workers in the province of KwaZulu-Natal (KZN) [7]. The Methods for Improving Reproductive Health in Africa (MIRA) diaphragm study showed a 73% HSV-2 prevalence in Durban, with 41% of these women co-infected with Human Immunodeficiency Virus (HIV) [8].

Two decades of observational and biological research supports the theory that HSV-2 increases the risk of HIV-1 acquisition up to four fold [1, 2, 4, 9–11] and increases the risk of HIV-1 transmission and infectiousness [4, 9, 12]. A meta-analysis demonstrated that HSV-2 seropositivity was associated with a HIV acquisition risk ratio of 2.7 in men and 3.1 in women. HSV-2 infection has also been associated with increased incidence of other STIs, such as *Neisseria gonorrhoeae (*NG)*, Treponema pallidum* (TP) *and Trichomonas vaginalis* (TV) [11]. This would emphasize the need to test HSV-2 control strategies as a means to reduce the transmission not only of HIV, but also of other STIs [11].

In the KZN province, HIV prevalence among sexually active women attending three clinics was estimated to be 40% [13]. There are many reasons as to why the epidemic is spreading uncontrollably in this region; socio-economic conditions and specific population dynamics (such as patterns of sexual networking, levels of condom use and STI infections), are known to be important determinants in the spread of HIV infection [14]. Poor socioeconomic conditions often result in women engaging in commercial sex activity. Poverty is also known to result in male migrant labour, resulting in men being away from their primary sexual partners for extended periods of time. This often leads to men having multiple sexual partners [14, 15]. In KZN, a high rate of multiple, concurrent and age-discrepant sexual relationships also contribute to the spread of HIV in the region. In this largely patriarchal society, gender inequality and the inability of women to negotiate condom use further contribute to these factors [14, 15]. Given the relationship between HSV-2 and HIV infection, it is important to ascertain the factors that put HIV uninfected women at risk of HSV-2 infection in regions of high HIV endemicity.

The current study estimates the prevalence and risk factors associated with HSV-2 infection among women who tested HIV-1 negative in a cohort from an HIV prevention trial. We also estimated the incidence of HIV-1 and HSV-2 infection among these women during study follow-up. These data provide an opportunity to gain further insight into the HSV-2 epidemic in the KZN region and to inform the design of future HIV/STI prevention strategies.

## Methods

The Microbicide Development Programme (MDP) 301 study (2005–2009), was a multicenter, international, double-blind, randomized, placebo-controlled trial assessing the safety and efficacy of 0.5 and 2% PRO 2000/5 gels for the prevention of vaginally acquired HIV infection [16, 17]. Women from six research centers (13 sites) in Africa were enrolled. However, since KwaZulu-Natal is considered the epicenter of the HIV epidemic, only women enrolled at the three Durban sites of the Medical Research Council (MRC) of South Africa research center were considered in this analysis. The protocol was approved by the Biomedical Research Ethics Committee based at the University of KwaZulu-Natal, the Medicines Control Council in South Africa and the US Food and Drug Administration. The eligibility criteria included: willingness and ability to give informed consent; HIV-seronegative status at screening; age 18 years or older; non-pregnant and not planning to become pregnant for the duration of the trial; willing to have regular speculum examinations and urinary pregnancy tests; willing to be tested for HIV infection; willingness to use study gel as advised; likely to be sexually active; and willingness to receive counseling about condoms. Women were excluded if they had any severe clinical, laboratory or gynecological abnormalities; had sex >14 times per week, and/or were unlikely to comply with the protocol. Women were followed up every 4 weeks for a total of 52 weeks. Women were provided with HIV testing and counseling, risk reduction education and diagnosis and treatment of STIs. Participants diagnosed with curable STIs were invited to bring their male partners to the study site for treatment or were provided with referral cards to access STI treatment at local clinics. Women received unrestricted supplies of condoms at all study visits and were counseled to use condoms during all sex acts. The primary efficacy endpoint of the MDP301 trial was HIV-1 infection, using a novel HIV confirmatory testing algorithm [18]. Briefly, the algorithm included parallel HIV-1 rapid tests at the clinics, with positive or discordant tests after enrolment prompting Enzyme Immunoassay testing at local laboratories and confirmation at a central laboratory in South Africa. The central laboratory analysed samples from the study visit at which the first positive rapid test result was obtained, in addition to previous study visits at which samples were obtained. At enrolment and at predefined study visits, additional procedures were carried out according to the study schedule. These included a clinical evaluation with genital examination, and the collection of additional specimens for the confirmation of the diagnosis of HIV (Enrolment, week 12, week 24, week 40 and week 52), HSV-2 (Enrolment, week 40 and week 52), syphilis (Enrolment, week 24 and week 52), and other infections [*N. gonorrhoeae* (NG), *C. trachomatis* (CT), *Trichomonas vaginalis* (TV), bacterial vaginosis and candida] at the Enrolment and week 24 visits. HSV-2 was assessed using the HerpeSelect 2 IgG ELISA (FOCUS Diagnostics, California, USA); Focus index values of ≥3.5 were considered HSV-2 seropositive [sensitivity of 92% (95% confidence interval (CI) 82–98) and specificity of 91% (95% CI 79–98)] [19]. Chlamydial and gonococcal infections were assessed using amplicor swabs collected during the genital examination by using nucleic acid amplification assays (COBAS Amplicor, Roche Molecular Diagnostics, Pleasanton, CA, USA). Baseline and confirmatory syphilis testing was conducted on sera using the rapid plasma reagin (RPR) (BD Macrovue) and *Treponema palladium* haemagglutination (TPHA) (Fujibrio) methods respectively. Testing for TV was conducted using sterile swabs with the In-Pouch-TV test kit [16].

The primary outcome measure of this analysis was HSV-2 seropositivity at baseline and the risk factors associated with HSV-2 seropositivity. In addition, we assessed the risk factors associated with HSV-2 incidence during study follow-up. The following variables were considered: age (in quartiles), education level (secondary school completed vs not completed), number of sex acts in last week (less than 3 vs more than 3), number of lifetime sexual partners (1 partner vs 2 or more partners), employment (yes/no), religion (Christian vs other), age at first sex act (15 and younger vs 16 and over), contraception use [none (reference), hormonal contraception, condoms, other (tubal ligation, rhythm method)] and being infected with another STI (CT, NG, TV or syphilis). Univariate and multivariate logistic regression models were used to identify the correlates of HSV-2 infection at screening. The multivariate model was constructed using a forward stepwise method. We used data driven cut point methods, such as quartiles, to categorize the continuous age variable. Regarding the number of sex acts per week, the data driven method was used in such a way that each level had enough observations; therefore they will not be over- or under-representative of the respective groups. Central tendency measures, such as the median, and dispersion measures [interquartile ranges (IQRs)] were as follows: the median for overall age was 27 (IQR 22–37), with the median for HSV-2 negative women being 23 (IQR 20–29) and the median for HSV-2 positive women being 31 (IQR 23-39) (*p* value for rank test is <0.001); the median for number of sex acts per week was three for the study population overall (IQR 2–4), with a median of 3 (IQR 2–4) for both the HSV-2 positive and negative populations (p value for rank test was 0.511). Independent predictors of HSV-2 infection were assessed using Cox proportional hazard regression model. A univariate logistic regression was conducted first; risk factors with a p value of <0.10 were considered as candidates for the multivariate model. We used a forward stepwise technique to build the final model. All the factors with a p value of <0.05 were retained in the final multivariate model. Variables were considered statistically significant if p < 0.05. HIV incidence was defined as the time from enrolment to seroconversion and/or censoring at the end of study follow-up on the basis of a discrete time scale determined by a woman’s study visits at 12, 24, 40 and 52 weeks. The time of seroconversion was defined as the time of the initial positive HIV test result. If one or more visit between the last negative HIV tests and the first positive tests were missed, the time of seroconversion was presumed to be the visit comprising the midpoint between these two time points. Similarly, the time to an HSV-2 positive result after enrolment (tested for at weeks 40 and 52) or censoring was used to calculate the HSV-2 incidence rate during study follow-up. All analyses were conducted using STATA 12.0 (College Station, Texas, USA).

## Results

A total of 2236 HIV-negative women were screened and enrolled into the MDP 301 trial at clinical research sites in Durban, South Africa. The HSV-2 prevalence at baseline in this cohort of women was 65%. The HIV incidence among HSV-2 negative women was 5.2 per 100 person years (PY), compared to 8.7 per 100 PY for women that were HSV-2 positive at baseline (p < 0.001, log rank test). The HSV-2 incidence during study follow-up was 22.3 per 100 PY (95% CI 20.4–24.3).

Table 1 presents the risk factors for being diagnosed with HSV-2 at baseline. In the univariate analysis, the risk of HSV-2 infection increased with age [23–27 years old odds ratios (OR) 1.76, 95% confidence intervals (CI) 1.39–2.2; age 28–37 years old OR 3.40, 95% CI 2.67–4.33; age >38 years old OR 6.72, 95% CI 5.06–8.92, respectively]. However, engaging in their first sex act at an age younger than 15 years old did not significantly increase a woman’s risk of being HSV-2 positive (OR 1.33, 95% CI 0.88–2.02), but having two or more lifetime sex partners did significantly increase risk of being HSV-2 positive (OR 1.27, 95% CI 1.06–1.55). In addition to age, being unemployed (OR 1.85, 95% CI 1.43–2.4) or having less than high school education (OR 1.78, 95% CI 1.47–2.14) increased the odds of a woman being HSV-2 positive at baseline. The type of contraception used by women also had an impact on HSV-2 positivity; women using “tubal ligation, rhythm, intrauterine devices or traditional” forms of contraception were more at risk of being HSV-2 positive (OR 1.745, 95% CI 1.09–2.8) than women using no contraception, whereas women using hormonal contraception or condoms were less at risk of being HSV-2 positive (OR 0.6, 95% CI 0.44–0.89; OR 0.515, 95% CI 0.36–0.74, respectively) than women using no contraception. When considering STIs, infection with CT did not increase the odds of being HSV-2 positive, while being infected with GC, TV and/or Syphilis did (OR 1.89, 95% CI 1.07–3.31, OR 1.51, 95% CI 1.09–2.09 and OR 4.62, 95% CI 1.97–10.81 respectively); although all STIs significantly increased the risk of being HSV-2 positive at screening in the multivariate analysis. These risk factors remained in the multivariate analysis, except for religion, number of sex acts per week and hormonal contraception (as these were not found to be significant in the univariate analysis).

**Table 1:** Risk factors for being diagnosed with HSV-2 at baseline

Variable	Overall number (% of group)	Univariate analysis		Multivariate analysis	
	N = 2236	Odds ratio (95% CI)	p value	Odds ratio (95% CI)	p value
Age*					
<23	660 (29.5)	1		1	
23–27	484 (21.6)	1.76 (1.39–2.23)	*0.001*	1.75 (1.37–2.23)	*0.001*
28–37	564 (25.2)	3.40 (2.67–4.33)	*0.001*	3.24 (2.50–4.20)	*0.001*
38+	528 (23.6)	6.72 (5.06–8.92)	*0.001*	6.08 (4.44–8.32)	*0.001*
Employed/income					
Yes	1842 (82.4)	1.0		1.0	
No	394 (17.6)	1.85 (1.43–2.36)	*0.001*	1.43 (1.10–1.87)	*0.008*
Religion					
Other	1346 (60.2)	1.0		1.0	
Christian	890 (39.8)	1.09 (0.91–1.30)	0.334	–	–
Education					
≥High school	1550 (69.3)	1.0			
<High school	686 (30.7)	1.78 (1.47–2.14)	*0.001*	1.24 (1.01–1.52)	*0.037*
Age at first sex act (years)					
>15	2123 (94.9)	1.0			
<15	113 (5.1)	1.33 (0.88–2.02)	0.175	–	–
Sex acts per week**					
<3	1282 (57.3)	1.0			
3+	954 (42.7)	0.99 (0.83–1.18)	0.934	–	–
Sexual partners					
1 partner	606 (27.1)	1.0			
2 + partners	1630 (72.9)	1.27 (1.06–1.55)	*0.011*	–	–
STI					
*C. trachomatis*	244 (10.9)	1.07 (0.81–1.42)	0.624	1.51 (1.11–2.04)	*0.007*
*N. gonorrhea*	71 (3.2)	1.89 (1.07–3.31)	*0.027*	2.37 (1.31–4.29)	*0.004*
Syphilis	56 (2.5)	4.62 (1.97–10.81)	*0.001*	2.88 (1.20–6.94)	*0.018*
*T. vaginalis*	200 (8.9)	1.51 (1.09–2.09)	*0.013*	1.50 (1.06–2.12)	*0.024*
Contraception					
None	185 (8.3)	1.0			
Other^a^	228 (10.2)	1.75 (1.09–2.81)	*0.021*	–	–
Hormonal	1196 (53.49)	0.63 (0.44–0.89)	*0.008*	–	–
Condoms	627 (28.0)	0.52 (0.36–0.74)	*0.001*	–	–

*CI* confidence interval

^a^Includes tubal ligation, intrauterine devices and traditional methods

* Median (IQR) = 27 (23–37); p value for rank test was < 0.001

** Median (IQR) = 3 (2–4); p value for rank test was 0.511

Table 2 presents the predictors of HSV-2 incidence during study follow-up. Being over 38 years old was a significant predictor for HSV-2 incidence [hazard ratio (HR) 1.57 (95% CI 1.23–2.00)], which was sustained in the multivariate analysis [HR 1.71 (95% CI 1.34–2.19)]. Christianity was also a predictor of HSV-2 incidence in the study [HR 1.21 (95% CI 1.01–1.44)], although this was not sustained in the multivariate analysis. HIV seroconversion during study follow-up was a significant predictor for HSV-2 seroconversion in the univariate [HR 1.71 (95% CI 1.30–2.24)] and in the multivariate analysis [HR 1.84 (95% CI 1.40–2.42)]. Contrary to this, a woman who had an STI at baseline or during the study was not at greater risk of HSV-2 seroconversion. Similar to our HSV-2 prevalence data, women who used “other” methods of contraception were more at risk of becoming HSV-2 positive during the study. Another interesting observation was that pregnancy incidence increased a woman’s risk of HSV-2 infection, both in univariate [HR 1.25 (95% CI 0.93–1.67) (p < 0.133)] and multivariate analysis [HR 1.37 (95% CI 1.08–1.83) (p < 0.038)], although this was only statistically significant in the multivariate analysis.

**Table 2:** Predictors of incidence of HSV-2

Variable	Univariate analysis		Multivariate analysis	
	Hazard ratio (95% CI)	p value	Hazard ratio (95% CI)	p value
Age*				
<23	1		1	
23–27	1.18 (0.91–1.54)	0.213	1.21 (0.93–1.58)	0.155
28–37	1.20 (0.93–1.55)	0.157	1.27 (0.98–1.64)	0.066
38+	1.57 (1.23–2.00)	*<0.001*	1.71 (1.34–2.19)	*<0.001*
Employed/income				
Yes	1.03 (0.88–1.30)	0.805		
No	1			
Religion				
Other	1			
Christian	1.21 (1.01–1.44)	*0.040*	–	–
Education				
≥High school	1			
<High school	1.11 (0.91–1.35)	0.289		
Age at first sex act (years)				
>15	1			
<15	1.11 (0.76–1.64)	0.586	–	–
STI				
*C. trachomatis*	1.07 (0.80–1.43)	0.663	–	–
*N. gonorrhea*	0.76 (0.49–1.18)	0.221	–	–
Syphilis	1.23 (0.66–2.31)	0.510	–	–
*T. vaginalis*	0.97 (0.71–1.32)	0.835	–	–
Contraception				
None	1			
Other^a^	1.39 (1.08–1.82))	*0.012*	–	–
Hormonal	0.86 (0.59–1.25)	0.416	–	–
Condom	0.93 (0.75–1.14)	0.481	–	–
Biological factors				
STI (s) baseline	1.04 (0.84–1.29)	0.705	–	–
STI (s) incidence	1.13 (0.90–1.42)	0.314	–	–
HIV incidence	1.71 (1.30–2.24)	*<0.001*	1.84 (1.40–2.42)	*<0.001*
Pregnancy incidence	1.25 (0.93–1.67)	0.133	1.37 (1.02–1.83)	*0.038*

*CI* confidence interval

^a^Includes tubal ligation, intrauterine devices and traditional methods

* Median (IQR) = 27 (23–37); p value for rank test was <0.001

## Discussion

Although the HSV-2 prevalence in this study is high, it is similar to previous studies from developing countries [4, 5] and other South African studies [6, 7, 20, 21]. This study was conducted at three urban clinical research sites in Durban. Interestingly, the MIRA study, which was conducted by the MRC at one rural and one semi-rural clinical research site in the Durban area 2 years prior to the MDP 301 trial, showed a HSV-2 prevalence of 73% (median age was 26; IQR 22–34) [8]. This higher prevalence in rural/semi-rural areas of Durban could be explained by several factors. Since women would have less access to jobs in a rural area, they may engage in more transactional sex; rural areas have poorer access to healthcare, including STI treatment and free condoms; and women in rural areas may have had less opportunity for education than those women from urban areas, which may increase their risk of acquiring HSV-2. The HSV-2 incidence during study follow-up is staggering and is much higher than previously seen in other South African studies. The HSV-2 incidence seen in our study compares to HSV-2 studies in Kenya and Uganda among female sex workers [22]. However, another cohort of Kenyan women, who considered themselves to be at high risk of HIV infection but were not sex workers, had a similar incidence of 22.1 per 100 person-years [23], while a study at an STI clinic in Alabama showed women had a HSV-2 incidence of 20.5 per 100 woman-years [24]. Women participating in these HIV prevention trials usually consider themselves at high risk of HIV infection, which could explain this high incidence of HSV-2 due to risky sexual behavior. The HIV incidence seen in this cohort of women was comparable to the HIV incidence among HSV-2 positive women in the MIRA trial [20].

Previous analyses of HSV-2 risk factors have shown that women whose sexual debut was at an age younger than 16 were at higher risk of being HSV-2 positive, however this was not significant in our cohort [8, 25]. Similar to other studies, our results showed that women who had not attended high school were at greater risk of having acquired HSV-2 [26] and this is possibly due to high school attendance influencing the structure of sexual networks [27]. Unemployment also put a woman at risk of being HSV-2 positive, most likely due to unemployed women being dependent on their partner for income, which may result in them being unable to negotiate safe sex with partners. Additionally, unemployment can drive women to engage in transactional sex. Consistent with other studies, the risk of being HSV-2 positive increased with age [25, 28]. This is most likely due to an increased number of sexual partners and increased years of sexual activity with age (both risk factors of HSV-2 infection [25, 26, 28]). Women presenting for this clinical trial in Durban, who were infected with an STI were significantly more likely to be co-infected with HSV-2 than women in the trial that did not have an STI, although syphilis showed the greatest risk of HSV-2 co-infection. We did note some instances of co-infections with STIs. Thirty three women were co-infected with TV and CT, while 31 women were found to be co-infected with NG and CT (data not shown). The relationships between HSV-2 and other STIs were expected, since it is widely known that there is increased incidence of STIs with HSV-2 infection, specifically NG, TV, and *T. pallidum* infections [11, 25, 28]. However, the same was not observed when considering HSV-2 incidence during study follow-up. This is not surprising, as women would have been treated for their STIs as soon as they were diagnosed, according to the study protocol requirements, which could explain why having an STI is a risk factor prior to study enrolment, but not during the study. The increased risk of HSV-2 infection we experienced in pregnant women is not surprising, since both are as a result of participating in unprotected sex.

Another study looking at HSV-2 in an HIV prevention trial in rural/semi-rural Durban sites found that women older than 25, having less than high school education, being single or cohabiting, being a Christian, young age of sexual debut, more than four lifetime partners, giving birth to more than one child and co-infection with NG put women at an increased risk of being HSV-2 positive [8]. Together with this study, our analysis provides us with a detailed set of risk factors suited to this region that has high HSV-2 and HIV infection.

The use of hormonal contraceptives has recently come under the spotlight for possibly increasing the risk of HIV-1 acquisition. However, our results showed that a woman on hormonal contraception was less likely to be HSV-2 infected at baseline in addition to being at less risk of HSV-2 infection during study follow-up. The link between hormonal contraception and HIV infection is a highly debated one, with the majority of experts agreeing that hormonal contraception does not increase risk of HIV infection, while the use specifically of depot medroxyprogesterone acetate may pose a risk of infection [29, 30]. In fact, some studies have shown a decreased risk of HIV infection with progesterone only pills and combined oral contraception [adjusted hazard ratio (HR_a_)  =  0.86, 95% confidence interval (CI) 0.32, 1.78] [31]. Of note with our data is that hormonal contraception includes injectable contraception and oral contraceptives, which have not shown an increased risk in HIV acquisition. One study evaluating the effect of hormonal contraception on risk of HSV-2 infection showed that injectable contraception did not increase this risk [32]. Further studies need to be conducted to confirm these findings.

HSV-2 has emerged as the most prevalent STI, and since it is most often asymptomatic, unrecognized and, most importantly, incurable, it puts individuals at increased risk of acquiring other STIs in addition to HIV. Given the high prevalence and incidence of HSV-2 infection reported here, screening for symptomatic HSV-2 among high risk populations should be incorporated into HIV and STI screening and treatment packages. Women also need to be encouraged to seek treatment for STIs, including symptomatic HSV-2. Young women need to be educated about the risk of HSV-2 infection, its impact on HIV and STI acquisition, and how to prevent HIV and STI acquisition. Despite condoms being an effective method of HIV prevention, they have only been shown to reduce the transmission of HSV-2 by 30% [33]. Understanding the prevalence and risk factors for common causes of ulcerative genital disease in the general population would inform current STI syndromic management and HIV testing strategies in high HIV prevalence regions. Lastly, it is vital that prevention approaches integrate the management of both bacterial and viral STIs for young women in these high prevalence regions.

Several limitations need to be considered when interpreting our results: this data was taken from the responses of women participating in a large, randomized, controlled, HIV prevention trial, and therefore may have limited representativeness. Women participating in these trials often participate because they perceive themselves to be at high risk of HIV infection and therefore may not represent the general population. The trial protocol also limits the sex and age of participants. In addition to this, we have not considered the effects of unmeasured characteristics, such as cultural differences (e.g. polygamy), poverty, commercial sex and multiple or concurrent sex partners in our findings. As this analysis was limited to data that was collected as part of an HIV prevention trial, the independent variables included are limited. No socioeconomic or behavioral data was collected from the male partners of these women. Due to the fact that we could not determine the characteristics of women who did not present for enrollment into this clinical trial, we must be cautious when extrapolating these results to the population as a whole. However, these results provide valuable information about HIV negative women presenting to participate in a HIV prevention trial. These results could also indicate that there is a much higher prevalence of HSV-2 in the HIV-1 positive population in KZN.

## Abbreviations

CI: confidence interval
CT: *C. trachomatis*
HIV: human immunodeficiency virus
HR: hazard ratio
HSV-2: Herpes Simplex Virus 2
IQR: interquartile ranges
KZN: KwaZulu-Natal
MDP: Microbicide Development Programme
MIRA: Methods for Improving Reproductive Health in Africa
MRC: Medical Research Council
NG: *N. gonorrhoeae*
OR: odds ratio
PY: person years
SSA: sub-Saharan Africa
STI: sexually transmitted infection
TPHA: *Treponema palladium* haemagglutination
TV: *Trichomonas vaginalis*

## References

[1] Celum C, Levine R, Weaver M, Wald A (2004). Genital herpes and human immunodeficiency virus: double trouble. Bull World Health Organ.

[2] Looker KJ, Garnett GP, Schmid GP (2008). An estimate of the global prevalence and incidence of herpes simplex virus type 2 infection. Bull World Health Organ.

[3] Suazo PA, Tognarelli EI, Kalergis AM, González PA (2015). Herpes simplex virus 2 infection: molecular association with HIV and novel microbicides to prevent disease. Med Microbiol Immunol.

[4] Paz-Bailey G, Ramaswamy M, Hawkes SJ, Geretti AM (2007). Herpes simplex virus type 2: epidemiology and management options in developing countries. Sex Transm Infect.

[5] Bastien S, Mason-Jones A, De Koker P, Mmbaga E, Ross D, Mathews C (2012). Herpes simplex virus type 2 infection as a biomarker for sexual debut among young people in sub-Saharan Africa: a literature review. Int J STD AIDS.

[6] Jewkes R, Nduna M, Levin J, Jama N, Dunkle K, Puren A, Duvvury N (2008). Impact of stepping stones on incidence of HIV and HSV-2 and sexual behaviour in rural South Africa: cluster randomised controlled trial. BMJ.

[7] Ramjee G, Williams B, Gouws E, Van Dyck E, De Deken B, Karim SA (2005). The impact of incident and prevalent herpes simplex virus-2 infection on the incidence of HIV-1 infection among commercial sex workers in South Africa. J Acquir Immune Defic Syndr.

[8] Abbai NS, Wand H, Ramjee G (2015). Socio-demographic and behavioural characteristics associated with HSV-2 sero-prevalence in high risk women in KwaZulu-Natal. BMC Res Notes.

[9] Sobngwi-Tambekou J, Taljaard D, Lissouba P, Zarca K, Puren A, Lagarde E, Auvert B (2009). Effect of HSV-2 serostatus on acquisition of HIV by young men: results of a longitudinal study in Orange Farm, South Africa. J Infect Dis.

[10] Lingappa JR, Kahle E, Mugo N, Mujugira A, Magaret A, Baeten J, Bukusi EA, Cohen CR, Katabira E, Ronald A (2009). Characteristics of HIV-1 discordant couples enrolled in a trial of HSV-2 suppression to reduce HIV-1 transmission: the partners study. PLoS ONE.

[11] Kaul R, Nagelkerke NJ, Kimani J, Ngugi E, Bwayo JJ, MacDonald KS, Rebbaprgada A, Fonck K, Temmerman M, Ronald AR (2007). Prevalent herpes simplex virus type 2 infection is associated with altered vaginal flora and an increased susceptibility to multiple sexually transmitted infections. J Infect Dis.

[12] Celum C, Wald A, Lingappa JR, Magaret AS, Wang RS, Mugo N, Mujugira A, Baeten JM, Mullins JI, Hughes JP (2010). Acyclovir and transmission of HIV-1 from persons infected with HIV-1 and HSV-2. N Engl J Med.

[13] Nel A, Mabude Z, Smit J, Kotze P, Arbuckle D, Wu J, van Niekerk N, van de Wijgert J (2012). HIV incidence remains high in KwaZulu-Natal, South Africa: evidence from three districts. PLoS ONE.

[14] Harrison A, Cleland J, Frohlich J (2008). Young people’s sexual partnerships in KwaZulu-Natal, South Africa: patterns, contextual influences, and HIV risk. Stud Fam Plann.

[15] Garnett GP, Anderson RM (1993). Factors controlling the spread of HIV in heterosexual communities in developing countries: patterns of mixing between different age and sexual activity classes. Philos Trans R Soc Lond B Biol Sci.

[16] McCormack S, Ramjee G, Kamali A, Rees H, Crook AM, Gafos M, Jentsch U, Pool R, Chisembele M, Kapiga S (2010). PRO2000 vaginal gel for prevention of HIV-1 infection (Microbicides Development Programme 301): a phase 3, randomised, double-blind, parallel-group trial. Lancet.

[17] Nunn A, McCormack S, Crook AM, Pool R, Rutterford C, Hayes R (2009). Microbicides Development Programme: design of a phase III trial to measure the efficacy of the vaginal microbicide PRO 2000/5 for HIV prevention. Trials.

[18] Jentsch U, Lunga P, Lacey C, Weber J, Cairns J, Pinheiro G, Joseph S, Stevens W, McCormack S (2012). The implementation and appraisal of a novel confirmatory HIV-1 testing algorithm in the Microbicides Development Programme 301 Trial (MDP301). PLoS ONE.

[19] Delany-Moretlwe S, Jentsch U, Weiss H, Moyes J, Ashley-Morrow R, Stevens W, Mayaud P (2010). Comparison of focus HerpesSelect and Kalon HSV-2 gG2 ELISA serological assays to detect herpes simplex virus type 2 antibodies in a South African population. Sex Transm Infect.

[20] de Bruyn G, Shiboski S, van der Straten A, Blanchard K, Chipato T, Ramjee G, Montgomery E, Padian N (2011). The effect of the vaginal diaphragm and lubricant gel on acquisition of HSV-2. Sex Transm Infect.

[21] Auvert B, Ballard R, Campbell C, Caraël M, Carton M, Fehler G, Gouws E, MacPhail C, Taljaard D, Van Dam J (2001). HIV infection among youth in a South African mining town is associated with herpes simplex virus-2 seropositivity and sexual behaviour. AIDS.

[22] Rajagopal S, Magaret A, Mugo N, Wald A (2014). Incidence of herpes simplex virus type 2 infections in Africa: a systematic review. Open Forum Infect Dis.

[23] Okuku HS, Sanders EJ, Nyiro J, Ngetsa C, Ohuma E, McClelland RS, Price MA, Graham SM (2011). Factors associated with herpes simplex virus type 2 incidence in a cohort of human immunodeficiency virus type 1-seronegative Kenyan men and women reporting high-risk sexual behavior. Sex Transm Dis.

[24] Gallo MF, Warner L, Macaluso M, Stone KM, Brill I, Fleenor ME, Hook EW, Austin HD, Lee FK, Nahmias AJ (2008). Risk factors for incident herpes simplex type 2 virus infection among women attending a sexually transmitted disease clinic. Sex Transm Dis.

[25] Watson-Jones D, Weiss HA, Rusizoka M, Baisley K, Mugeye K, Changalucha J, Everett D, Balira R, Knight L, Ross D (2007). Risk factors for herpes simplex virus type 2 and HIV among women at high risk in northwestern Tanzania: preparing for an HSV-2 intervention trial. J Acquir Immune Defic Syndr.

[26] Wald A (2004). Herpes simplex virus type 2 transmission: risk factors and virus shedding. Herpes.

[27] Hargreaves JR, Bonell CP, Boler T, Boccia D, Birdthistle I, Fletcher A, Pronyk PM, Glynn JR (2008). Systematic review exploring time trends in the association between educational attainment and risk of HIV infection in sub-Saharan Africa. AIDS.

[28] Van de Laar M, Termorshuizen F, Slomka M, Van Doornum G, Ossewaarde J, Brown D, Coutinho R, Van den Hoek J (1998). Prevalence and correlates of herpes simplex virus type 2 infection: evaluation of behavioural risk factors. Int J Epidemiol.

[29] Morrison CS, Chen PL, Kwok C, Baeten JM, Brown J, Crook AM, Van Damme L, Delany-Moretlwe S, Francis SC, Friedland BA (2015). Hormonal contraception and the risk of HIV acquisition: an individual participant data meta-analysis. PLoS Med.

[30] Polis CB, Phillips SJ, Curtis KM, Westreich DJ, Steyn PS, Raymond E, Hannaford P, Turner AN (2014). Hormonal contraceptive methods and risk of HIV acquisition in women: a systematic review of epidemiological evidence. Contraception.

[31] McCoy SI, Zheng W, Montgomery ET, Blanchard K, van Der Straten A, de Bruyn G, Padian NS (2013). Oral and injectable contraception use and risk of HIV acquisition among women in sub-Saharan Africa. AIDS.

[32] Noguchi L, Hillier S, Richardson B, Chirenje Z, Balkus J, Piper J, Marrazzo J (2015). O18. 2 Injectable progestin contraception and acquisition of hsv-2 infection among South African women participating in the voice trial. Sex Transm Infect.

[33] Martin ET, Krantz E, Gottlieb SL, Magaret AS, Langenberg A, Stanberry L, Kamb M, Wald A (2009). A pooled analysis of the effect of condoms in preventing HSV-2 acquisition. Arch Intern Med.

